# CXXC1 is not essential for normal DNA double-strand break formation and meiotic recombination in mouse

**DOI:** 10.1371/journal.pgen.1007657

**Published:** 2018-10-26

**Authors:** Hui Tian, Timothy Billings, Petko M. Petkov

**Affiliations:** The Jackson Laboratory, Bar Harbor, ME 04609, United States of America; Cornell University, UNITED STATES

## Abstract

In most mammals, including mice and humans, meiotic recombination is determined by the meiosis specific histone methytransferase PRDM9, which binds to specific DNA sequences and trimethylates histone 3 at lysine-4 and lysine-36 at the adjacent nucleosomes. These actions ensure successful DNA double strand break formation and repair that occur on the proteinaceous structure forming the chromosome axis. The process of hotspot association with the axis after their activation by PRDM9 is poorly understood. Previously, we and others have identified CXXC1, an ortholog of *S*. *cerevisiae* Spp1 in mammals, as a PRDM9 interactor. In yeast, Spp1 is a histone methyl reader that links H3K4me3 sites with the recombination machinery, promoting DSB formation. Here, we investigated whether CXXC1 has a similar function in mouse meiosis. We created two *Cxxc1* conditional knockout mouse models to deplete CXXC1 generally in germ cells, and before the onset of meiosis. Surprisingly, male knockout mice were fertile, and the loss of CXXC1 in spermatocytes had no effect on PRDM9 hotspot trimethylation, double strand break formation or repair. Our results demonstrate that CXXC1 is not an essential link between PRDM9-activated recombination hotspot sites and DSB machinery and that the hotspot recognition pathway in mouse is independent of CXXC1.

## Author summary

Meiotic recombination increases genetic diversity by ensuring novel combination of alleles passing correctly to the next generation. In most mammals, the meiotic recombination sites are determined by histone methyltransferase PRDM9. These sites are proposed to become associated with the chromosome axis with the participation of additional proteins and undergo double strand breaks, which are repaired by homologous recombination. In budding yeast, Spp1 (ortholog of CXXC1) binds to methylated H3K4 and connects these sites with the chromosome axis promoting DSB formation. However, our data suggest that even though CXXC1 interacts with PRDM9 in male germ cells, it does not play a crucial role in mouse meiotic recombination. These results indicate that, unlike in yeast, a recombination initiation pathway that includes CXXC1 could only serve as a non-essential pathway in mouse meiosis.

## Introduction

Meiotic recombination ensures production of fertile gametes with a correct haploid chromosome number and genetic diversity [1]. In most of mammals, the meiotic recombination sites are restricted to 1–2 kb regions, termed recombination hotspots, locations of which are determined by the DNA binding histone methyltransferase PRDM9 [2–4]. Recombination initiates when PRDM9 binds to hotspots sequences with its zinc finger domain and trimethylates histone 3 at lysine 4 (H3K4me3) and lysine 36 (H3K36me3) resulting in formation of a nucleosome-depleted region [2–6], probably by the action of nucleosome motors [7]. DNA double strand breaks (DSB) are created at the nucleosome-depleted regions of activated hotspots [8–11], and eventually repaired as either crossovers or non-crossover conversions. Cytological staining for several proteins associated with DSB processing in early meiotic prophase show that, from the earliest time of their detection, DSB are associated with a proteinaceous structure known as chromosome axis [12–15]. We have previously shown that PRMD9 is associated, but not directly interacting, with chromosome axis elements such as phosphorylated REC8 (pREC8) and SYCP3 in spermatocytes [16]. Efficient H3K4 trimethylation at hotspots is crucial for normal DSB formation and repair that occurs on the chromosome axis [17], even though PRDM9 binding presumably occurs on the open chromatin loops [18]. However, we currently do not have detailed knowledge of the proteins and molecular mechanisms participating in hotspot association with the chromosome axis.

In *Saccharomyces cerevisiae*, which has no PRDM9, the PHD zinc finger protein Spp1, a member of the COMPASS complex (Complex associated with Set1, the protein catalyzing trimethylation of histone 3 at lysine-4), acts as a histone H3K4 methyl reader and promotes meiotic DSB formation at the existing H3K4me3 sites, such as promoters [19, 20]. Spp1 is predominantly located on the chromosome axes and connects H3K4me3 sites with the axis protein Mer2 to stimulate Spo11 dependent DSB formation [19, 20]. Recent study showed that Spp1 function in tethering DSB sites to chromosome axes and ensuring efficient DSB formation is independent of its function as a COMPASS complex member [21].

CxxC finger protein 1 (CXXC1, also known as CFP1 and CGBP) is an ortholog of *S*. *cerevisiae* Spp1 in mammals [21]. In somatic cells, CXXC1 binds to both unmethylated CpGs and SETD1, which is required for trimethylation of H3K4 at CpG islands [22]. CXXC1 is crucial for embryonic stem cell maintaining and development [23, 24]. Knocking out *Cxxc1* in mice results in lethality at the early embryonic stages [25]. We have reported that CXXC1 interacts with PRDM9 in yeast two-hybrid assay and *in vitro* [16]. This interaction has recently been confirmed by another group, which also reported that CXXC1 interacts with the chromosome axis element IHO1 by yeast two-hybrid assay [26]. IHO1 is considered to be the ortholog of yeast Mer2 and is known to be essential for ensuring efficient DSB formation [27], therefore it is possible that CXXC1-IHO1 interaction serves the same function in mammalian meiosis as their orthologs Spp1-Mer2 in yeast. However, the function of CXXC1 in mammalian meiosis has not been characterized so far. It has been unclear whether CXXC1 binds to PRDM9 in germ cells and whether it participates in meiotic recombination initiation in any way, either as a partner of PRDM9 or as a methyl reader of H3K4me3/H3K36me3 marks that PRDM9 imposes at the nucleosomes surrounding the recombination hotspots.

Here we confirmed that CXXC1 is co-expressed with PRDM9 and indeed interacts with it in spermatocytes. To address whether and how CXXC1 functions in meiotic recombination, we created two *Cxxc1* conditional knockout mouse models and deleted *Cxxc1* in all germ cells and in late spermatogonia just before the onset of meiosis. In both models, loss of CXXC1 did not affect normal meiotic recombination process. Our study demonstrates that the presence of CXXC1 in mouse meiosis is not essential, and unlike its *S*. *cerevisiae* ortholog Spp1, CXXC1 does not appear to be a key factor for the DSB formation.

## Results

### CXXC1 interacts with PRDM9 in spermatocytes

**Fig 1:**
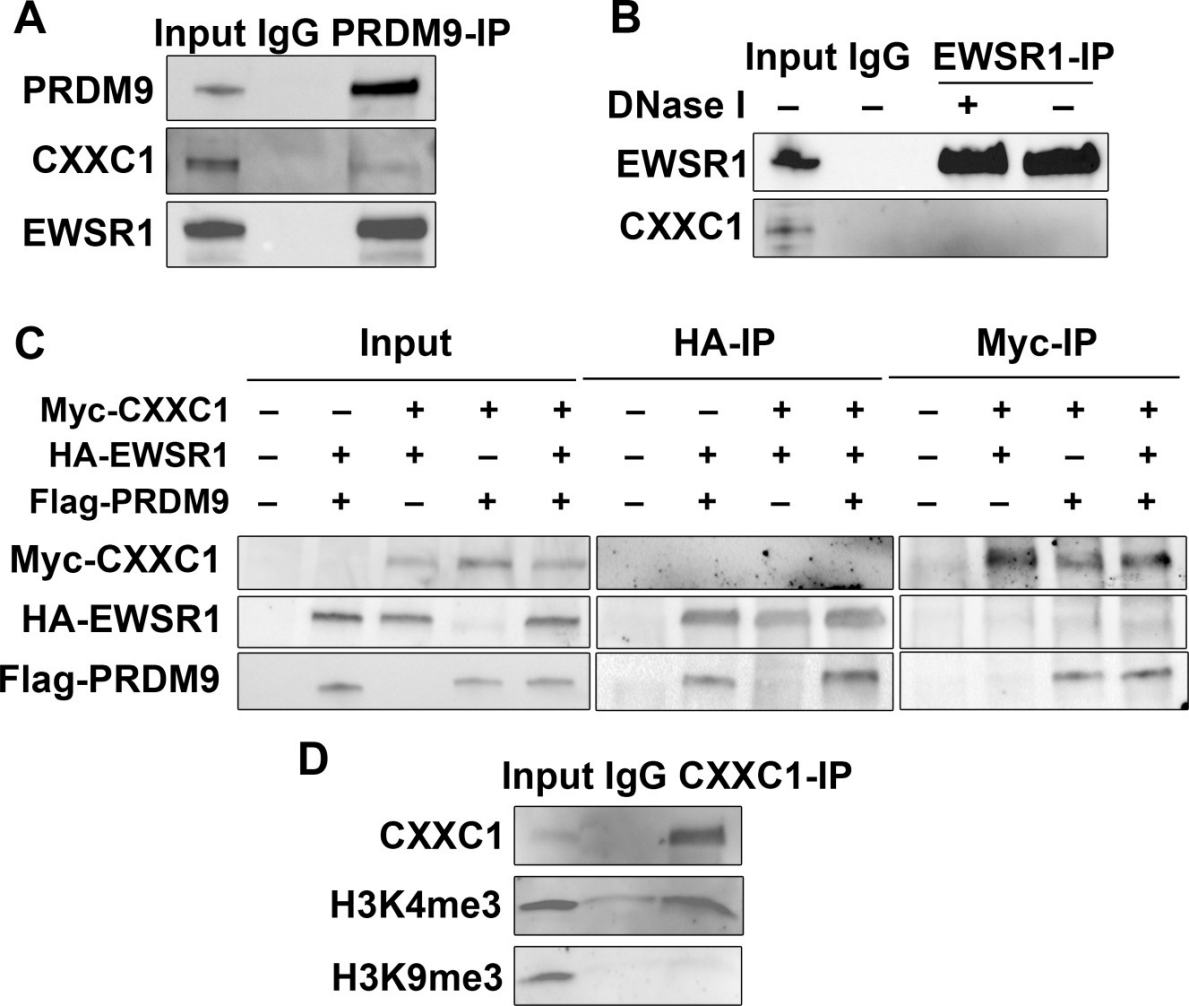
CXXC1 interacts with PRDM9 in spermatocytes. (A) PRDM9 interacts with CXXC1 and EWSR1 in spermatocytes. Co-IP with PRDM9 from 14 dpp B6 testicular extract. Staining for each protein is indicated on left. In each blot, lane 1- input; lane 2 –co-IP with non-immune IgG; lane 3 –co-IP with antibody against PRDM9. (B) EWSR1 does not interact with CXXC1 in spermatocytes. Co-IP with EWSR1 from 14 dpp B6 testicular extract. Staining for EWSR1 and CXXC1. In each blot, lane 1- input; lane 2 –co-IP with non-immune IgG; lane 3 –co-IP with anti-EWSR1 after DNase I treatment; co-IP with anti-EWSR1 without DNase I treatment. (C) CXXC1 interacts with PRDM9 but not with EWSR1 in cell culture. Myc tagged CXXC1, HA tagged EWSR1 and Flag tagged PRDM9 were transfected into HEK293 cells. Input controls (left panel), co-IP with HA antibody (middle panel), and IP with Myc antibody (right panel). In each row, staining for the indicated proteins is shown. (D) CXXC1 binds to H3K4me3 but not to H3K9me3. Co-IP with CXXC1 from 14 dpp B6 testicular extract. Top row, staining for CXXC1; middle row, staining for H3K4me3; bottom row, staining for H3K9me3.

We tested whether CXXC1 interacts with PRDM9 *in vivo* by co-immunoprecipitation (co-IP) from spermatocytes isolated from 14 dpp B6 testis using antibody against PRDM9 [16]. We found that CXXC1 indeed interacts with PRDM9 in spermatocytes (Fig 1A). However, the interaction was not as strong as with PRDM9’s predominant interactor EWSR1 (Fig 1A) [16], which raised the possibility that the interaction between CXXC1 and PRDM9 could be mediated by stronger PRDM9 interactors. To test whether this is the case, we performed co-IP with EWSR1 and did not detect any interaction with CXXC1 in testicular extract (Fig 1B). To further test the interactions between the three proteins, we co-expressed Myc-tagged mouse CXXC1, HA-tagged EWSR1 and Flag-tagged PRDM9 proteins in human embryonal kidney 293 (HEK293) cells, and performed co-IP with antibodies against HA or Myc tags. Both EWSR1 and CXXC1 immunoprecipitated PRDM9 under these conditions, but there was no interaction between CXXC1 and EWSR1 in the presence or absence of PRDM9 (Fig 1C).

Several reports have shown that Spp1, the yeast ortholog of CXXC1, binds to H3K4me3 and tethers H3K4-trimethylated recombination hotspots to the chromosome axis [19–21]. To test whether CXXC1 binds to H3K4me3 in mouse spermatocytes, we performed CXXC1 co-IP from 14-dpp B6 testicular extract. Indeed, we detected CXXC1 interaction with H3K4me3 but not with the closed chromatin mark H3K9me3 (Fig 1D).

These results indicate that CXXC1 and EWSR1 form separate complexes with PRDM9. They also indicate that although CXXC1 interacts with PRDM9 *in vivo*, it is not a predominant interactor of PRDM9, and that their interaction could be mediated by other proteins such as histone 3 trimethylated at lysine 4.

### CXXC1 is present in nuclei of all meiosis stages

**Fig 2:**
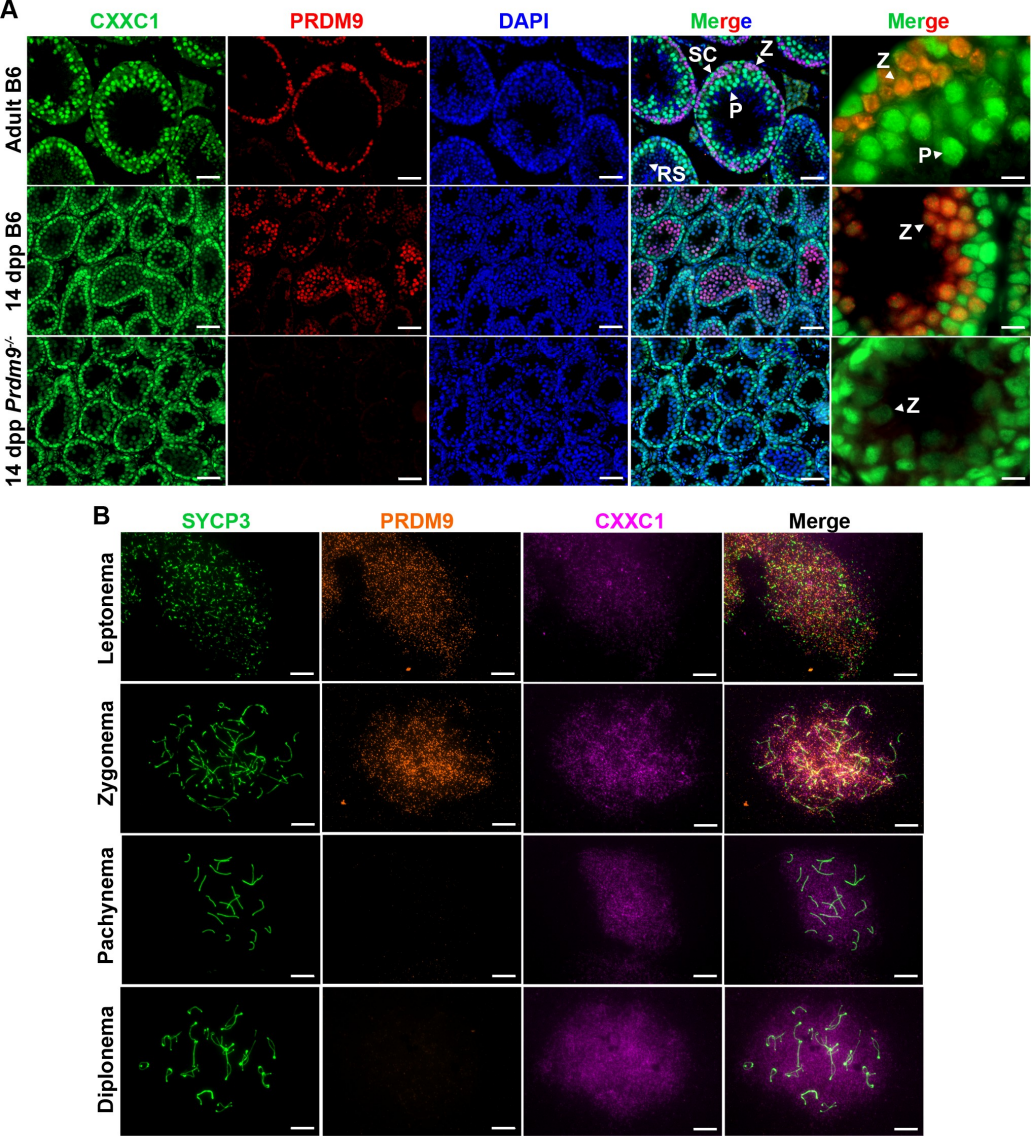
CXXC1 is expressed in the spermatocytes in the presence or absence of PRDM9. (A) Immunofluorescence staining for CXXC1 and PRDM9 in adult B6, 14-dpp B6 and 14-dpp *Prdm9*^*-/-*^ seminiferous tubule cross sections. Green, CXXC1; red, PRDM9; blue, DAPI. SC, Sertoli cell; Z, zygonema; P, pachynema; RS, round spermatid. Scale bars, first 4 columns: 50 μm, last column: 10 μm. (B) Immunofluorescence staining for CXXC1 and PRDM9 on chromosome spreads from adult B6. Green, SYCP3; orange, PRDM9; magenta, CXXC1. Scale bars, 10 μm.

In seminiferous tubules of mouse testis, CXXC1 is expressed in both germ cells and Sertoli cells (Fig 2A, top panel). CXXC1 showed high expression in spermatogonia, low expression in leptonema and zygonema, and then again high expression in pachynema and diplonema, decreasing to undetectable levels in spermatids (Fig 2A, top panel).

Previous reports showed that PRDM9 is present only in leptonema and zygonema during meiosis [28]. Double staining for PRDM9 and CXXC1 showed co-expression of these two proteins in nuclei from stage X seminiferous tubules (Fig 2A, top panel) and in 14-dpp (days post partum) testis (Fig 2A, middle panel), when the majority of spermatocytes are at leptotene and zygotene stages. Since CXXC1 interacts with PRDM9 *in vivo* (Fig 1A), we performed CXXC1 staining in *Prdm9* knockout mouse testis (*Prdm9*^*-/-*^) to determine whether CXXC1 localization could be affected by the absence of PRDM9. In this mutant, CXXC1 showed the same localization pattern as in controls (Fig 2A, bottom panel). The pattern of PRDM9 and CXXC1 in leptonema and zygonema was further confirmed by chromosome spreads, where CXXC1 showed diffused signal over the entire nuclear region from leptonema through diplonema (Fig 2B).

These results show that CXXC1 and PRDM9 are both present in leptonema and zygonema nuclei, and that CXXC1 expression and localization are not affected by the presence or absence of PRDM9.

### Male *Cxxc1* knockout mice are fertile

**Fig 3:**
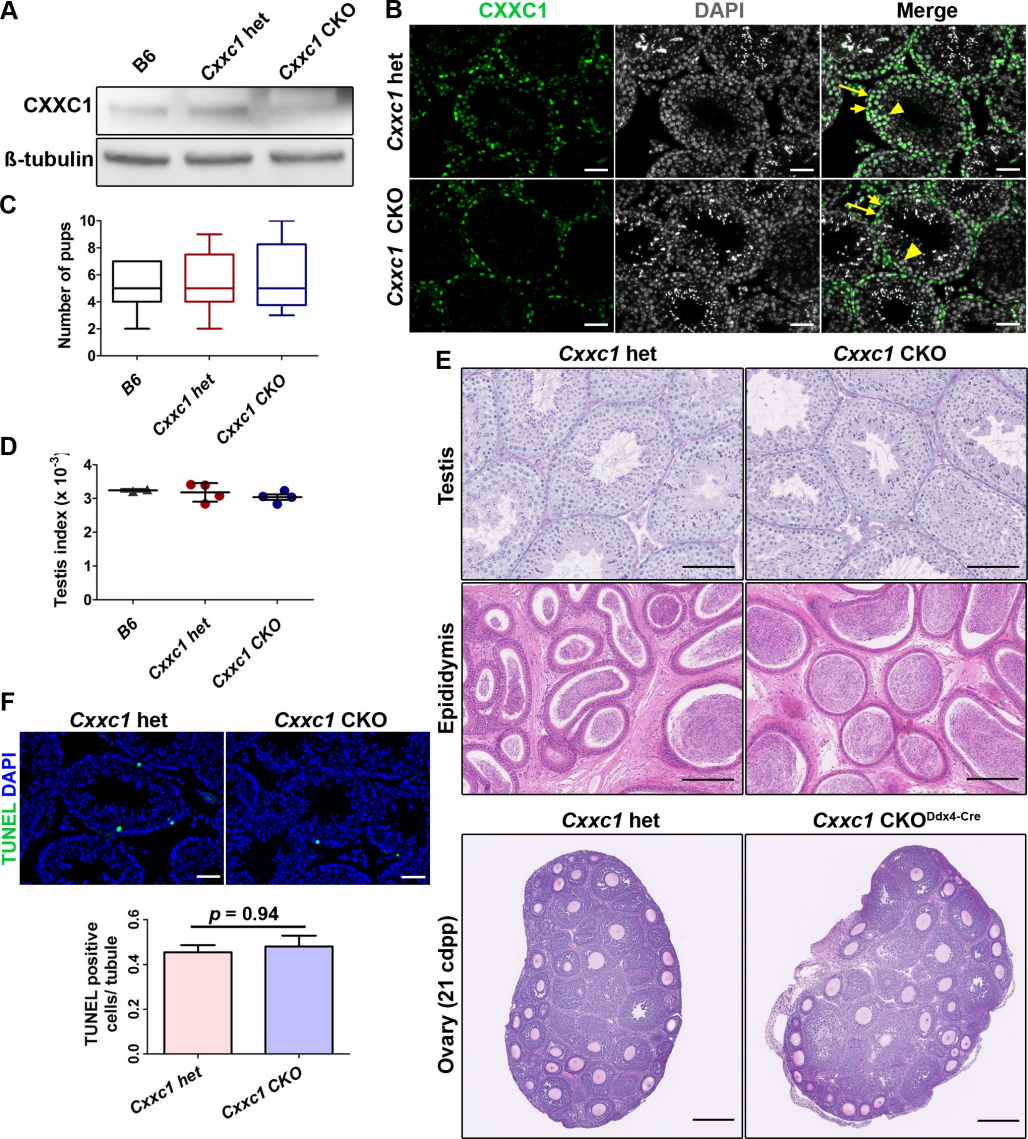
Knocking out CXXC1 does not affect male fertility or testis histology. (A) CXXC1 is depleted in Stra8-cre CKO testes. Western blot of CXXC1 from adult B6, *Cxxc1* het and CKO testicular extract. ß-tubulin was used as internal loading control. (B) CXXC1 is present in Sertoli cells but not in spermatocytes of Stra8-cre CKO mice. Immunostaining of CXXC1 on *Cxxc1* het and CKO seminiferous tubule cross sections. Green, CXXC1; grey, DAPI. Long arrows, Sertoli cells; short arrows, spermatogonia; arrowhead, spermatocytes. Scale bar, 50 μm. (C) No change in fertility tests in CKO mice compared to B6 and *Cxxc1* heterozygous controls. The number of viable pups for each genotype is shown. (D) Testis index (testis weight/body weight) is not changed in CKO mice compared to B6 and *Cxxc1* heterozygous controls. (E) Normal histology of testis, epididymis and ovary is observed in both *Cxxc1* control and CKO mice. Top panels, PAS staining of seminiferous tubule sections; scale bar, 100 μm. Middle panels, H&E staining of epididymis sections; scale bar, 200 μm. Bottom panels, H&E staining of 21 dpp ovary sections, Scale bar, 250 μm. Left panels, het control; right panels, *Cxxc1* CKO with Stra8-Cre in male mice, and Ddx4-Cre in females. (F) No increased apoptosis is observed in testes of CKO mice. TUNEL staining in *Cxxc1* het and CKO. Top panels, scale bar, 50 μm. Bottom panels, the apoptotic cell number is quantified as TUNEL positive cell number per seminiferous tubule. Data represent as mean ± SD, *p* = 0.94 by Student *t-*test.

To test whether CXXC1 is involved in spermatogenesis, we generated a conditional knockout (CKO) model using CRISPR/Cas9 to insert loxP sites flanking exon 2 and 3 of *Cxxc1* in C57BL/6J. The strategy of obtaining the CKO mutants is shown on S1 Fig. We bred late spermatogonia-specific knockout mice (*Cxxc1*^*loxP/Δ;Stra8-iCre*^, hereafter *Cxxc1* CKO) by crossing the *Cxxc1*^*loxP/loxP*^ mice with *Stra8-iCre* mice [29] and germ cell-specific knockout mice (*Cxxc1*^*loxP/Δ;Ddx4-Cre*^, hereafter *Cxxc1* CKO^Ddx4-Cre^) with *Ddx4-Cre* mice [30]. Western blot confirmed that in knockout testes, the protein level of CXXC1 is reduced (Fig 3A). CXXC1 was absent in spermatocytes of the CKO, but present in spermatogonia and Sertoli cells in CKO testes (Fig 3B, short and long arrows) and only in Sertoli cells in *Cxxc1* CKO^Ddx4-Cre^ (S2A Fig, long arrows).

We performed fertility test with two *Cxxc1*-deleted CKO mice of each model. To our surprise, in both models the male mice were fertile and produced similar number of viable progeny compared to the heterozygous (het) and B6 controls (Figs 3C and S2B). Testis index (testis weight/body weight) was the same in CKO as in het and wild type B6 controls (Fig 3D). Histology of testis and epididymis from CKO mice showed no detectable spermatogenesis defects (Figs 3E and S2C). No increased apoptosis in germ cells was detected using TUNEL assay (Figs 3F and S2D).

In contrast, *Cxxc1* germ cell-specific knockout female mice with *Ddx4-*Cre (reduced protein level shown in S1E Fig) were sterile—no viable pups were produced from homozygous knockout *Cxxc1* CKO^Ddx4-Cre^ mating test, while the heterozygous control (*Cxxc1*^*loxP/+;Ddx4-Cre*^) mating produced normal number of pups (5.3 ± 1.7). However, the histology of CKO ovaries from 21 dpp and adult female mice both showed normal ovary morphology and follicle formation (Figs 3E and S2F). Thus, their sterility is most likely due to early embryonic developmental deficiency caused by the lack of maternal genome activation at zygotic stage as reported before [31] and not meiotic defects.

### Expression and function of PRDM9 remains normal in *Cxxc1* CKO

**Fig 4:**
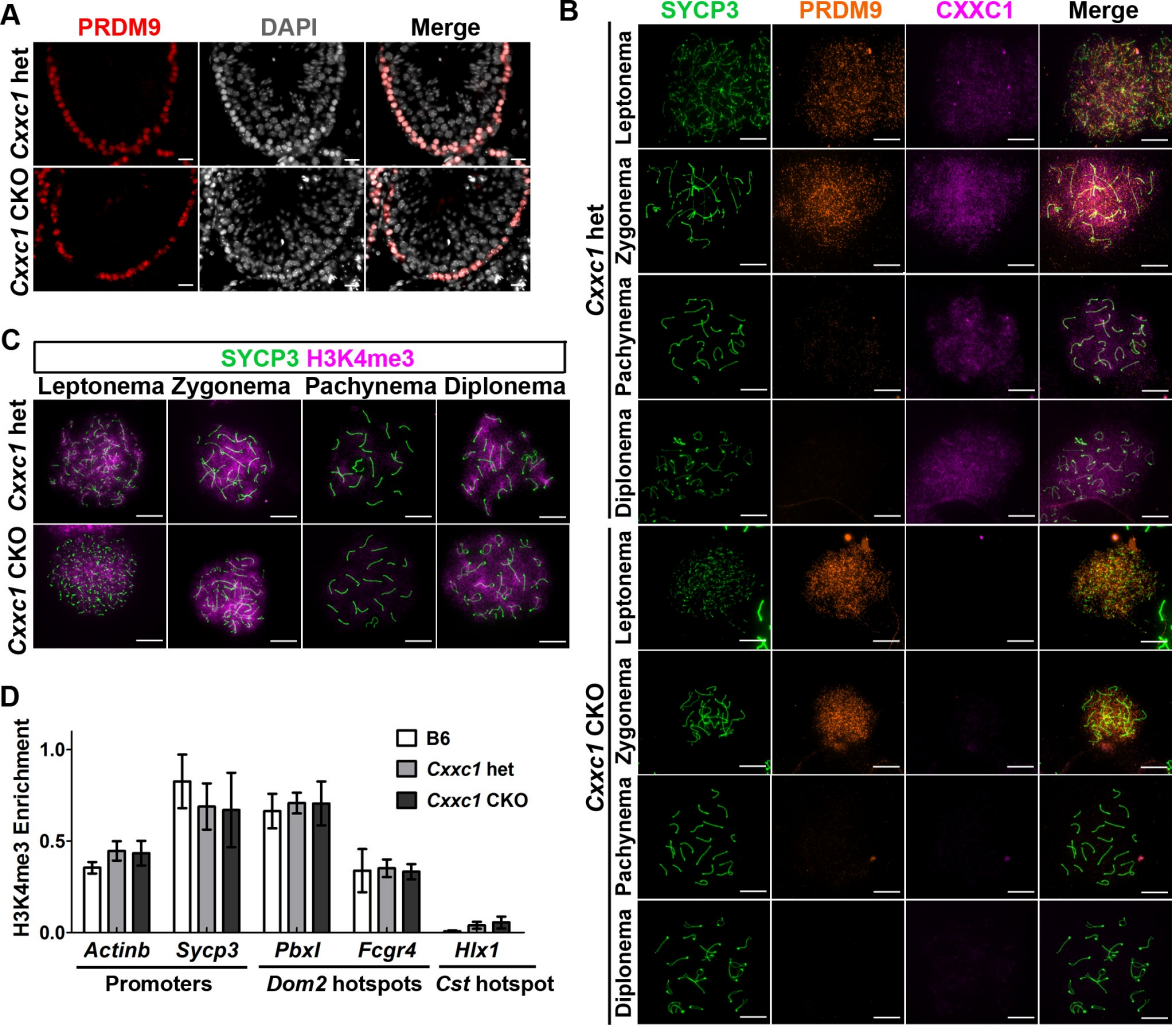
PRDM9 expression and catalytic function are not impaired in *Cxxc1* CKO. (A) Immunostaining of PRDM9 shows unchanged pattern in adult CKO seminiferous tubules compared to the heterozygous control. Red, PRDM9; grey, DAPI. Scale bar, 20 μm. (B) PRDM9 and SYCP3 expression patterns are not changed in CXXC1 CKO chromosome spreads compared to the heterozygous control. Co-immunostaining of CXXC1 and PRDM9 on chromosome spreads from adult *Cxxc1* het and CKO mice. Green, SYCP3; orange, PRDM9; magenta, CXXC1. First 4 rows, het control; last 4 rows, *Cxxc1* CKO. Scale bar, 10 μm. (C) Meiosis progression occurs normally in *Cxxc1* CKO testes. Immunostaining of H3K4me3 in adult *Cxxc1* het and CKO chromosome spreads. Green, SYCP3; magenta, H3K4me3. Scale bar, 10 μm. (D) Testis-specific gene expression is not changed in *Cxxc1* CKO testes. H3K4me3 ChIP-qPCR with chromatin isolated form *Cxxc1* het and CKO mice. Promoter regions form *Actinb* and *Sycp3*, *Dom2* hotspots *PbxI* and *Fcgr4* were amplified. *Cst* hotspot *HlxI* was used as a negative control. Bars present mean ± SD of three biological replicates.

We further tested whether CXXC1 affects the localization, the expression pattern, or the function of PRDM9. Localization of PRDM9 in seminiferous tubules was preserved in CKO (Figs 4A and S3A, right panel). In addition, the expression pattern of PRDM9 in leptonema and zygonema was not affected in the absence of CXXC1 (Fig 4B).

To test whether lack of CXXC1 affects PRDM9 methyltransferase function, we first compared H3K4me3 patterns in control and *Cxxc1* CKO mice. Both control and CKO chromosome spreads showed abundant H3K4me3 signal in leptonema and zygonema, lower signal in pachynema and increased signal in diplonema (Fig 4C). In addition, the H3K4me3 staining on cross sections of CKO testis showed no decrease (S3B Fig). These data indicate that the hotspot trimethylation and transcriptional activation in spermatocytes are not affected by the loss of CXXC1.

Second, we tested whether loss of CXXC1 affects PRDM9 binding and its methytransferase activity at individual hotspots by H3K4me3 ChIP-qPCR. We found that H3K4me3 enrichment at hotspots *PbxI* and *Fcgr4*, which are regulated by *Prdm9*^*Dom2*^, the *Prdm9* allele present in B6 mice, was not different in B6 control, *Cxxc1* heterozygous and CKO. We measured as a control the H3K4me3 enrichment at promoter regions of the housekeeping gene *Actinb* and the meiosis specific gene *Sycp3*, which are not PRDM9-dependent. These were not changed as well (Fig 4D).

These results suggest that loss of CXXC1 does not affect PRDM9 expression, binding to hotspots, or its catalytic function. Therefore, CXXC1 is not required for PRDM9-dependent hotspot activation.

### Double strand breaks occur normally at PRDM9 dependent sites in the absence of CXXC1

**Fig 5:**
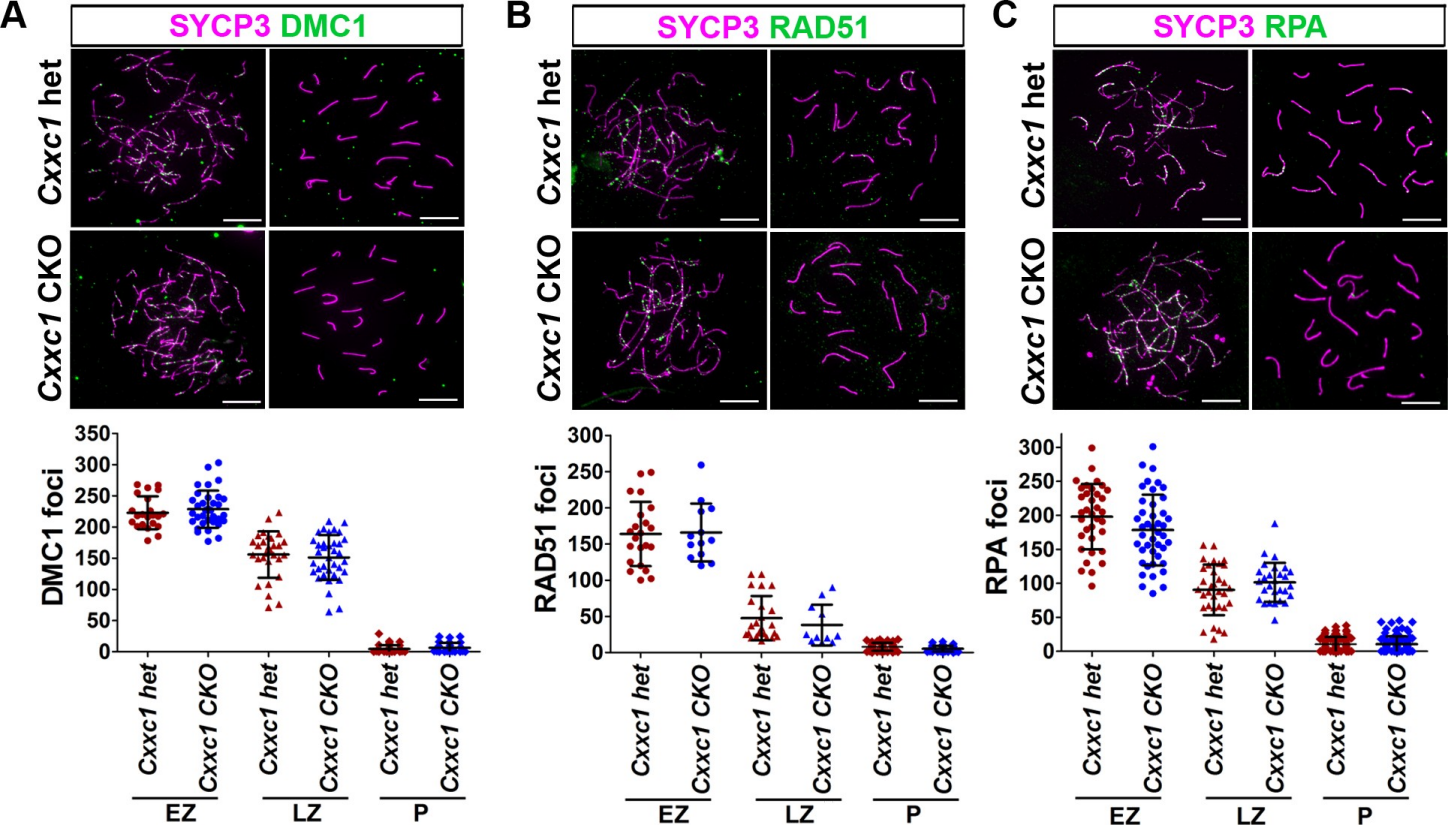
DSB number is not affected in *Cxxc1* CKO. The DSB number was determined by three markers reflecting different stages of their processing. (A) DMC1 staining on *Cxxc1* control and CKO chromosome spread. Bottom panels, distribution plot of DMC1 foci in early zygotene (n = 22 in het, n = 34 in CKO), late zygotene (n = 29 in het, n = 36 in CKO) and pachytene (n = 36 in het, n = 26 in CKO) spermatocytes. (B) RAD51 staining on *Cxxc1* control and CKO chromosome spread. Bottom panels, distribution plot of RAD51 foci in early zygotene (n = 22 in het, n = 13 in CKO), late zygotene (n = 24 in het, n = 11 in CKO) and pachytene (n = 52 in het, n = 36 in CKO) spermatocytes. (C) RPA staining on *Cxxc1* control and CKO chromosome spread. Bottom panels, distribution plot of RPA foci in early zygotene (n = 35 in het, n = 43 in CKO), late zygotene (n = 33 in het, n = 28 in CKO) and pachytene (n = 89 in het, n = 92 in CKO) spermatocytes. For A-C, two individuals per genotype were measured. Bars represent mean ± SD. Scale bar, 10 μm.

To test whether lack of CXXC1 affects DSB formation process, we next determined the number, position and activities of DSBs in the *Cxxc1* CKO. During DSB formation, the single stranded DNA tail is initially coated by the replication protein A (RPA), and then RPA is gradually replaced by the RecA family members RAD51 and DMC1 [32–35]. When measured by the number of foci of DMC1 (Figs 5A and S4A), RAD51 (Figs 5B and S4B) and RPA (Figs 5C and S4C) in early and late zygonema, these numbers in *Cxxc1* CKO were not statistically different from control samples (Fig 5A, 5B and 5C, lower panels). Also, we did not detect increased DMC1, RAD51 or RPA foci in CKO pachynema (Figs 5A, 5B and 5C and S4A, S4B and S4C). These data indicate that the number of DSB per meiosis and DSB repair process is not affected in the loss of CXXC1.

**Fig 6:**
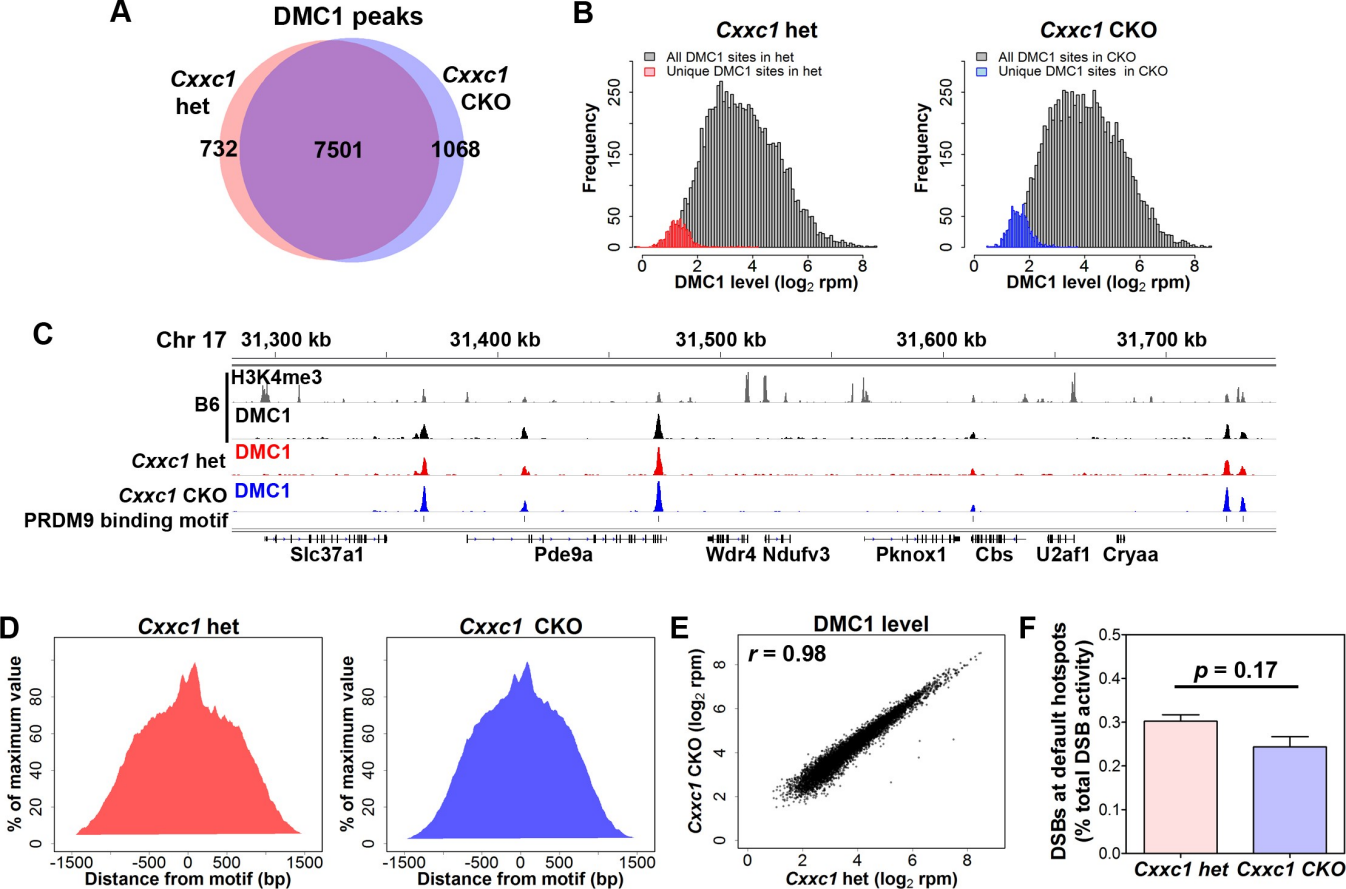
DSB occur at PRDM9 dependent sites in *Cxxc1* CKO. ChIP-seq data determining the number and positioning of DSB sites in CKO and heterozygous controls. (A) Venn diagram of DMC1 peak number in *Cxxc1* het and CKO. 7,501 peaks are shared in *Cxxc1* het and CKO. (B) Frequency distribution plot of DMC1 activity in *Cxxc1* het (left panel) and CKO (right panel). All DMC1 peaks (n = 7,501) were shown in grey in both panels; unique peaks in het controls (n = 732) were shown in red; unique peaks in CKO samples (n = 1,068) were shown in blue. (C) Coverage profiles of published B6 H3K4me3 (grey), B6 DMC1 (black), *Cxxc1* het DMC1 (red) and CKO DMC1 (blue) from a representative region on chromosome 17. PRDM9 binding motif sites are shown in line 5. (D) Aggregation plot of DMC1 signal in het control (left) and CKO (right). The signal was normalized to the maximum signal. (E) Plot of activity of DSBs from *Cxxc1* CKO and control spermatocytes. Correlation coefficient *r* = 0.98. (F) Percentage of DMC1 activity in default sites contributes to total activity.

To determine whether the locations of DSB sites in *Cxxc1* CKO are affected, we performed ChIP-seq for DMC1 [8, 9] (S5A Fig). We detected 8,233 DMC1 peaks in control spermatocytes and 8,569 DMC1 peaks in CKO spermatocytes, in which 7,501 peaks are shared in heterozygous control and CKO samples (Fig 6A). We plotted the frequency distribution of DMC1 activity of the 732 unique peaks from control (Fig 6B, left) or 1068 CKO unique peaks (Fig 6B, right), and found that these virtually unique peaks were not unique, but had low DMC1 activity which prevented them from being detected by the peak calling algorithm. We found that 91.8% of shared DMC1 peaks (6,886 peaks), 78.8% of control unique peaks (577 peaks) and 84.3% of CKO unique peaks (900 peaks) contain a PRDM9 binding site at their centers (Fig 6C as an example). The aggregation plots also confirmed that DSBs contain PRDM9 binding motifs at their centers in both control and CKO spermatocytes (Fig 6D). Also, in the 615 shared, 155 het unique and 168 CKO unique DMC1 peaks which do not contain detectable PRDM9 binding motifs, only 18, 5 and 5 peaks, respectively, overlapped with transcription start sites. Therefore, unlike in *Prdm9* knockout mice [9], promoter sites are not predominantly used for DSB formation in *Cxxc1* CKO.

The activity of DMC1 signal in control and CKO is highly correlated (*r* = 0.98, Fig 6D), indicating the activity of DSB formation is not affected in the CKO spermatocytes. Furthermore, the activity of default DSB sites, which do not contain PRDM9 binding motif in the center, only contributes to 0.30% and 0.24% of total DMC1 activity in control and CKO samples, respectively (Figs 6E and S5B), similar as reported default DSB activity in male germ cells [36].

These data suggest that loss of CXXC1 does not affect DSB number or positions. Therefore, CXXC1 is not essential for PRDM9 dependent DSB initiation pathway.

### Meiotic DSB repair is normal in *Cxxc1* CKO

**Fig 7:**
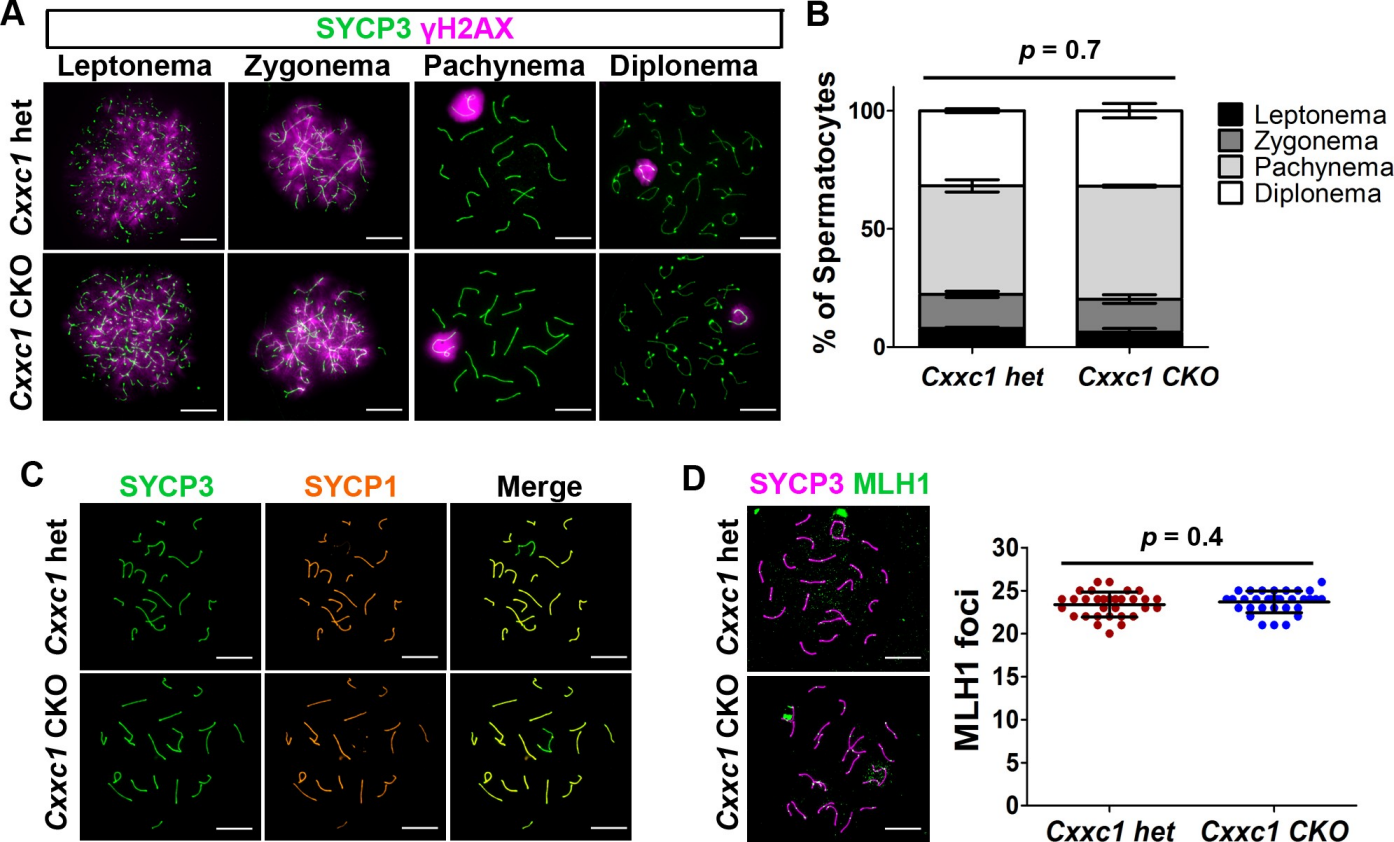
No major meiotic DSB repair or chromosome synapsis defects are observed in *Cxxc1* CKO testis. (A) Immunostaining of SYCP3 and γH2AX on adult *Cxxc1* het and CKO chromosome spreads. Green, SYCP3; magenta, γH2AX. Scale bar, 10 μm. (B) Spermatocyte stage proportion in adult *Cxxc1* het (n = 1,062 from two individuals) and CKO (n = 1,105 from two individuals) spermatocytes based on SYCP3/SYCP1/γH2AX staining. *p* = 0.7 by *Chi-square* test. (C) Immunostaining of SYCP3 and SYCP1 on adult *Cxxc1* het and CKO chromosome spreads. Green, SYCP3; orange, SYCP1. Scale bar, 10 μm. (D) Crossover number measured by MLH1 staining on chromosome spreads of adult *Cxxc1* het and CKO spermatocytes. Left, magenta, SYCP3; green, MLH1. Scale bar, 10 μm. Right, number of MLH1 foci per late pachynema in *Cxxc1* het (n = 32 from two individuals) and CKO (n = 33 from two individuals). Bars represent mean ± SD. *p* = 0.4 by Student’s t-test.

To further investigate whether the DSB repair process and chromosomal synapsis are impaired in CKO, we used staining for phosphorylated H2AX (γH2AX), which marks unrepaired DNA lesions and sex body in pachynema, to test for the processing of recombination repair. The pattern of γH2AX staining was not changed in CKO compared to the heterozygous control spermatocytes, showing γH2AX signal throughout the nucleus in leptonema when DSBs occur, which was then restricted to the sex body in pachynema when the autosomal breaks are repaired (Figs 7A and S4D). We also measured spermatocyte proportion based on the staining, and did not detect difference in cell proportion (Figs 7B and S4E). These results indicate that the sex body formation and DSB repair are not affected in the *Cxxc1* CKO, and there is no major cell loss or arrest in CKO meiosis.

Co-staining of SYCP1 and SYCP3 confirmed normal synapsis in all autosomes in CKO spermatocytes at pachynema (Figs 7C and S4F). 98.5% of *Cxxc1* CKO pachynema with Stra8-Cre and 98.23% of CKO pachynema with Ddx4-Cre showed full synapsis, compared with 97.5% of fully synapsed pachynema in control (*p* = 0.15 and 0.91, respectively). Therefore, there is no increased chromosome asynapsis in the CKO spermatocytes compared to controls.

Finally, we examined whether loss of CXXC1 affects crossover resolution. Using MLH1 as a marker of crossover sites, we did not find any significant change of crossover number in the CKO spermatocytes compared to the het controls (Fig 7D).

Taken together, these results suggest that even though CXXC1 interacts with PRDM9 and H3K4me3 in spermatocytes, it is not required for PRDM9 binding at hotspots, their subsequent activation by PRDM9-dependent H3K4 trimethylation, DSB formation, repair, or crossover formation, and is therefore not essential for meiotic recombination events.

## Discussion

In this study, we demonstrate that CXXC1 interacts with PRDM9 in spermatocytes. However, this interaction does not seem to be important for any cell function necessary for germ cell development or recombination processes. The germ cell specific *Cxxc1* knockout male mice are fertile. In the knockout spermatocytes, the expression and function of PRDM9 are unchanged. The loss of CXXC1 does not affect DSB formation and repair, chromosome pairing and synapsis, and crossover numbers. Together, these results convincingly show that CXXC1 is not essential for normal meiotic recombination events and generally for spermatogenesis and oogenesis.

The yeast CXXC1 ortholog Spp1 is reported to be a key player in recombination by linking H3K4me3 sites with the chromosome axis and connecting them with the recombination protein Mer2 [19–21]. This suggested that CXXC1 might play similar function in mammalian meiosis. However, our results show that CXXC1 is not an essential player in mammalian recombination where PRDM9 controls the initial recognition and activation of recombination hotspots. In the absence of CXXC1, hotspot activation, axis integrity, DSB formation and crossover resolution occur normally, showing that DSB formation and recombination determination in most of mammals, which use the PRDM9 dependent pathway, differs from that in the budding yeast. In species with functional PRDM9, the function of the RMM complex consisting of orthologs of the yeast Rec114, Mei4 and Mer2 (REC114, MEI4 and IHO1 in mice) is still conserved [27, 37–39], and the association between hotspots and chromosome axis is crucial for efficient DSB formation [27, 40, 41]. However, the interaction between CXXC1 and IHO1 does not seem to play the same functional role as the one between Spp1 and Mer2 in yeast. One important difference is that in organisms that do not use PRDM9, DSB occur at H3K4me3 sites, whereas in those that use PRDM9, DSB occur at hotspots where surrounding nucleosomes are methylated at both H3K4 and H3K36 [5, 6]. This raises the likelihood that proteins with H3K36me3 methyl-reading activity, such as PWWP domain containing proteins [42], or with both H3K4me and H3K36me binding capability, such as Tudor domain containing proteins [43], might be involved in hotspot recognition in these species. Alternatively, activated hotspots may be recruited to the chromosome axis and DSB machinery without assistance of an H3K4me3/H3K36me3 reader. A recent study demonstrated that randomized DSBs induced by radiation in *Spo11* mutant spermatocytes are associated with chromosome axis and can successfully recruit DSB repair proteins such as DMC1/RAD51 complex [44]. Other direct PRDM9 interactors, such as EWSR1, EHMT2, CDYL [16], PIH1D1 [26] and CTCF [45], could also be involved in hotspot association with the chromosome axis.

An alternative, PRDM9-independent pathway, can explain the fraction of DSB detected at promoters in wild type mice, and all DSB in PRDM9 mutant mice [9, 46]. Although we did not detect any substantial reduction of PRDM9-independent hotspot activity in the absence of CXXC1, this pathway could still, to some degree, involve CXXC1 as part of the SETD1 complex, known to bind H3K4me3 at promoters, in a way similar to Spp1-Mer2 role in yeast meiosis. It is not an essential pathway in most organisms using PRDM9 as hotspot determinant, but might play a major role in those lacking PRDM9, such as canids [47–49], where recombination hotspots are enriched in CpG-rich regions with a preference for unmethylated CpG islands [9, 17, 47, 50], similar feature as CXXC1 binding sites [22, 51]. One recent report of a woman having no active PRDM9 but completely fertile suggests that this pathway may become activated and ensure proper recombination even in organisms using PRDM9 as a recombination regulator [52].

## Methods

### Ethics statement

The animal care rules used by The Jackson Laboratory are compatible with the regulations and standards of the U.S. Department of Agriculture and the National Institutes of Health. The protocols used in this study were approved by the Animal Care and Use Committee of The Jackson Laboratory (Summary #04008). Euthanasia for this study was done by cervical dislocation.

### Mouse models

All wild-type mice used in this study were in the C57BL/6J (B6) background. The conditional-ready mutants were produced by flanking exons 2 and 3 of *Cxxc1* with loxP sites using CRISPR-Cas9. The *Cxxc1* conditional knockout mice used in this study were produced by a two-step deletion scheme. Mice that harbor two conditional *Cxxc1* alleles (*Cxxc1*^*loxp/loxp*^) were mated to Tg(Sox2-cre)1Amc/J mice (stock #004783) to generate one *Cxxc1* allele deleted mice. The *Cxxc1* hemizygous mice (*Cxxc1*^*Δ/+*^) were mated to Tg(Stra8-icre)1Reb/J (stock #08208) and Tg(Ddx4-cre)1Dcas/KnwJ (stock #018980) to obtain *Cxxc1*^*Δ/+;Stra8-iCre*^ and *Cxxc1*^*Δ/+;Ddx4-Cre*^ mice. Those *Ewsr1*^*Δ/+;Stra8-iCre*^ and *Cxxc1*^*Δ/+;Ddx4-Cre*^ mice were then mated to homozygous *Cxxc1* loxp mice to generate heterozygous control mice (*Cxxc1*^*loxP/+;Stra8-iCre*^and *Cxxc1*^*loxP/+;Ddx4-Cre*^ designated as *Cxxc1* controls) or conditional knockout mice (*Cxxc1*^*loxP/Δ;Stra8-iCre*^and *Cxxc1*^*loxP/Δ;Ddx4-Cre*^ designated as *Cxxc1* CKO).

B6;129P2-*Prdm9*^*tm1Ymat*^/J mice (*Prdm9*^*-/-*^) have been previously described [53]. All animal experiments were approved by the Animal Care and Use Committee of The Jackson Laboratory (Summary #04008).

### Co-immunoprecipitation assays

The co-immunoprecipitation assays for PRDM9 and EWSR1 with testicular extract were carried out using our reported protocol [16]. Total protein was extracted from testes of twenty 14-dpp B6 mice homogenized in 1 ml of Pierce IP buffer (Thermo Fisher Scientific, 87787). 10% of extract was set apart as input. Co- immunoprecipitation was performed by incubating extract with protein G Dynabeads conjugated with antibodies against PRDM9 [18, 54] or guinea pig IgG overnight at 4°C. The beads were washed three times with 1 ml of Pierce IP buffer, eluted with 200 μl of GST buffer (0.2 M glycine, 0.1% SDS, 1% Tween 20, pH 2.2) for 20 min at room temperature and neutralized with 40 μl of 1 M Tris-HCl, pH 8. After heated at 95°C for 5 min, 10 μg of IP and input samples were then subjected to electrophoresis and western blotting for detection of PRDM9 (1:1000, custom made), EWSR1 (1:1000, Abcam, ab54708) and CXXC1 (1:1000, Abcam, ab198977). The co-IP experiment is performed in two replicates.

The co-immunoprecipitation assays for PRDM9, CXXC1 and EWSR1 in cell culture were carried out using our reported protocol [55]. The vector expressing the PRDM9, CXXC1 and EWSR1 proteins were constructed by cloning mouse *Prdm9*, *Cxxc1* and *Ewsr1* cDNA into pCEP4-Flag, pCMV-Myc and pCMV-HA vectors, respectively. 2.5 μg of plasmids were transfected into HEK293 cells by X-tremeGENE HP DNA Transfection Reagent (Roche, 6366244001) in 6-well plates. At 2 days after transfection, cells were harvested and mixed with 600 μl Pierce IP buffer. 10% of extract was set apart as input. Co-immunoprecipitation was performed by incubating extract with protein G Dynabeads conjugated with antibodies against HA (Sigma, H9658) or Myc (Sigma, M5546) overnight at 4°C. After washing the beads and eluting with GST buffer, 10 μg of IP and input samples were then subjected to electrophoresis and western blotting for detection of HA, Myc and Flag (Sigma, F1804). All the blots were processed together with the same exposure.

Co- immunoprecipitation for CXXC1 was performed similarly to those for PRDM9 and EWSR1 with the following changes. The seminiferous tubules were digested with liberase and the germ cells were isolated. Then, the nuclei were isolated by incubation germ cells in hypotonic lysis buffer (10 mM Tris-HCL pH 8.0, 1 mM KCl, 1.5 mM MgCl_2_) for 30 min at 4°C and spinning down at 10,000 *g* for 10 min. The nuclear extract was obtained by incubation with the nuclear lysis buffer (50 mM HEPES, pH 7.8, 3 mM MgCl_2_, 300 mM NaCl, 1 mM DTT and 0.1 mM PMSF), 5 U/μl DNaseI and 2 U/ μl TurboNuclease for 30 min at 4°C. 10% of extract was saved as input. The co-IP was perform by incubating extract with protein G Dynabeads conjugated with antibodies against CXXC1 (Abcam, ab198977) or guinea pig IgG overnight at 4°C. After wash and elution, the IP and input samples were then subjected to electrophoresis and western blotting for detection of CXXC1 (1:1000, Abcam, ab198977), H3K4me3 (1:1000, Millipore, #07–473) and H3K9me3 (1:1000, Active Motif, 39766).

### Measurement of testis index

Testicular weight and body weight of adult B6 (n = 3), *Cxxc1* het (n = 3) and CKO (n = 4) mice were measured. Testis index was calculated as testis weight/body weight. Student’s t-test was used to determine the statistical significance.

### Fertility test

Male fertility test was performed with 3 *Cxxc1* het control and 5 CKO male mice. Each mouse was mated with at least two B6 females for at least two to five month period. Female fertility test was performed with 2 *Cxxc1* control and 2 CKO female mice. Each one was mated with one B6 male for 3 month period. Litter size and viable pup number were recorded.

### Histology

Testis, epididymis, ovaries from adult or 21 dpp *Cxxc1* het control or CKO mice were dissected out. Testis and epididymis were fixed with Bouin’s solution, and ovaries were fixed in 2% PFA. and the tissues were embedded in paraffin wax, and sectioned at 5 μm. Sections of testis were stained with Periodic acid–Schiff–diastase (PAS), and section of epididymis and ovaries were stained with haematoxylin and eosin (H&E) using standard techniques.

### Chromosome spread cytology

The drying-down technique [56] was used for preparation of chromosome spreads from spermatocytes of 14-dpp and 8-weeks B6, *Cxxc1* control or CKO mice. Chromosome spread slides were immunolabeled with anti-PRDM9 (1:200), CXXC1 (1:1000), SYCP1 (1:300, Novus, NB300-229), SYCP3 (1:400, Novus, NB300-231), γH2AX (1:1000, Abcam, ab26350), DMC1 (1:200, Santa Cruz, sc-8973), RAD51 (1:200, Santa Cruz, H-92), RPA (1:300, Abcam, ab87272) or MLH1 (1:100, BD Pharmingen, 550838) antibodies.

### Immunofluorescence stainings

For protein immunolocalization on tissue sections, testicular tissues from 8 week old B6, *Prdm9*^*-/-*^, *Cxxc1* control and CKO mice were dissected out, fixed with 4% paraformaldehyde solution overnight, embedded in paraffin wax. 5-μm sections were prepared. For antigen retrieval, sections were heated in a microwave in Tris-EDTA buffer (10mM Tris, 1mM EDTA and 0.05% Tween 20, pH 9.0) for 10 min and cooled down to room temperature. Then, sections were treated with PBS containing 0.1% Triton X-100, blocked with 10% normal donkey serum, and stained with antibodies against PRDM9 (1:200), CXXC1 (1:1000) or H3K4me3 (1:1000, Millipore, #07–473).

### H3K4me3 chromatin immunoprecipitation and real-time PCR

Chromatin immunoprecipitation (ChIP) was performed as previously described [57]. Briefly, spermatocytes were isolated from 14-dpp B6, *Cxxc1* het and CKO spermatocytes, and crosslinked using 1% formaldehyde. Nuclei were isolated using hypotonic lysis buffer (10 mM Tris-HCL pH 8.0, 1 mM KCl, 1.5 mM MgCl_2_) and digested by MNase. The ChIP was done using antibody against H3K4me3. Real-time PCR was performed with purified ChIP DNA using Quantifast SYBR Green PCR Kit (Qiagen) Primer sequences used for real-time PCR are: *Pbx1*_F: AGAAACTGACATATGAAGGCTCA; *Pbx1*_R: GCTTTTGCTCCCTTAAACTGG; *Fcgr4*_F: CAAGGTGCATTCTTAGGAGAGA; *Fcgr4_*R: TTAATGCTTGCCTCACGTTC; *Hlx1*_F: GGTCGGTGTGAGTATTAGACG; *Hlx1*_R: GGCTACTATACCTTATGCTCTG; *Actinb*_promoter_F: GCCATAAAAGGCAACTTTCG; *Actinb*_promoter_R: TTTCAAAAGGAGGGGAGAGG; *Sycp3*_promoter_F: AAGGCGCCACAACCAAGG; *Sycp3*_promoter_F: TGCCTGGATGCCCAACTC.

### DMC1 ChIP-seq and data analysis

DMC1 ChIP was performed from spermatocytes of 8 weeks old *Cxxc1* het and CKO (Stra8-Cre) using an established method [58] in two replicates. The testes were cross-linked with 1% paraformaldehyde solution for 10 min, and then homogenized. After that, the nuclei were isolated, the chromatin was sheared to ~1000 bp by sonication and incubated with antibody against DMC1 overnight at 4°C, and then with protein G Dynabeads (Thermo Fisher Scientific, 10004D) for 4 hrs at 4°C. The beads were washed once with wash buffer 1 (0.1% SDS, 1% Triton X-100, 2 mM EDTA, 20 mM Tris-HCl, pH 8.0, 150 mM NaCl), wash buffer 2 (0.1% SDS, 1% Triton X-100, 2mM EDTA, 20mMTris-HCl, pH 8.0, 500 mM NaCl), then wash buffer 3 (0.25 M LiCl, 1% NP-40, 1mM EDTA, 10mMTris-HCl, pH 8.0, 1% Deoxycholic acid), and finally twice with TE buffer. The chromatin was eluted with dilution buffer (1% SDS, 0.1 M NaHCO_3_ pH 9.0) at 65°C for 30 min and then reverse-crosslinked by adding 200 mM NaCl and incubation overnight at 65°C. The libraries were then prepared according to the currently established method [58], and sequenced on an Illumina HiSeq 2500 platform, with 75 bp paired-end reads.

Fastq files for sequenced DMC1 libraries were trimmed using Trimmomatic (v0.32) and subsequently parsed for detection and selection of paired reads having homology at the 5’ and 3’ ends [9, 59] as established by protocols for single strand DNA enrichment to generate the files that contain only the detectable single strand reads, and then, these files were aligned to mm10 mouse genome using BWA (v.0.5.10-tpx). Bam files were parsed for detection and selection of reads containing true genomic sequence versus fill-in sequence at the homologous region. These reads were selected from the original paired-end fastq files, and then single-end fastq files were created that contained only the true genomic sequences of single strand DNA reads. All genomic data are available at NCBI Gene Expression Omnibus (GEO; http://www.ncbi.nlm.nih.gov/geo) under accession number GSE116336 (https://www.ncbi.nlm.nih.gov/geo/query/acc.cgi?acc=GSE116336). 1,688,630 and 1,349,516 aligned DMC1 reads in *Cxxc1* het controls, 1,590,925 and 1,961,645 aligned DMC1 reads in *Cxxc1* CKO spermatocytes were obtained from the two replicate libraries. The correlation between the two biological replicates in each experiment was high (*r* = 0.96 in *Cxxc1* het controls; *r* = 0.99 in *Cxxc1* CKO. S5A Fig); thus, the data from each pair of replicates were merged. The DMC1 activity was normalized to reads per million (rpm). Peak calling was performed using MACS (v.2.0.9) with a FDR value 0.01. PRDM9 dependent or default sites were determined using bedtools (v2.22.0) intersects compared with unknown PRDM9 binding sites (GEO number: GSE61613) [18]. Analyses for the aggregation plots were carried out using the ACT [60], of which parameters were: nbins = 500, mbins = 0, radius = 1500.

## Supporting information

S1 Fig Conditional knockout strategy of *Cxxc1*. Top line, wild type allele of *Cxxc1*; middle line, loxP allele of *Cxxc1* by flanking exon 2 and 3 with loxP sites; bottom line, deletion allele of *Cxxc1* after crossing the *Cxxc1*^*loxP/loxP*^ mice with Cre mice. Orange boxes, coding regions; blue boxes, 3 or 5 prime untranslated regions; black lines, intron regions; red triangles, loxP sites; red arrow, a stop codon generated by frame shift in the deletion allele; grey boxes, untranslated regions after stop codon in the deletion allele. Exon numbers are indicated as E1 to E15.
(TIF)

S2 Fig Knockout of CXXC1 in germ cells does not affect testis or ovary histology. (A) Immunostaining of CXXC1 on *Cxxc1* het and CKO^Ddx4-Cre^ seminiferous tubule cross sections. Green, CXXC1; grey, DAPI. Long arrows, Sertoli cells; short arrows, spermatogonia. Scale bar, 50 μm. (B) Fertility tests in *Cxxc1* het and CKO^Ddx4-Cre^ mice. The number of viable pups was shown. (C) PAS stating of seminiferous tubules in CKO^Ddx4-Cre^. Scale bar, 100 μm. (D) TUNEL staining (left) and quantification of apoptotic germ cells (right) in *Cxxc1* CKO^Ddx4-Cre^. Scale bar, 50 μm. Data represented as mean ± SD, *p* = 0.94 by Student *t-*test.(E) Western blot of CXXC1 with adult B6, *Cxxc1* het and CKO^Ddx4-Cre^ whole ovary extract. ß-tubulin was used as internal loading control. (F) H&E staining of secondary follicles in 4-month old *Cxxc1* het and CKO^Ddx4-Cre^. Scale bar, 50 μm.
(TIF)

S3 Fig PRDM9 and H3K4me3 patterns are not changed in *Cxxc1* CKO seminiferous tubule cross sections. (A) Immunostaining of PRDM9 in *Cxxc1* het and CKO^Ddx4-Cre^. Red, PRDM9; grey, DAPI. Scale bar, 20 μm. (B) Immunofluorescence staining of H3K4me3 on adult *Cxxc1* het and CKO with Stra8- and Ddx4-Cre seminiferous tubule cross sections. Magenta, H3K4me3; gray, DAPI. Scale bars: 50 μm.
(TIF)

S4 Fig DSB number is not affected in *Cxxc1* CKO^Ddx4-Cre^. The DSB number was determined by three markers reflecting different stages of their processing. (A) DMC1 staining on *Cxxc1* control and CKO chromosome spread. Lower panel, distribution plot of DMC1 foci in early zygotene (n = 20for each genotype), late zygotene (n = 25) and pachytene (n = 34) spermatocytes. (B) RAD51 staining on *Cxxc1* control and CKO chromosome spread. Lower panel, distribution plot of RAD51 foci in early zygotene (n = 8), late zygotene (n = 18) and pachytene (n = 53) spermatocytes. (C) RPA staining on *Cxxc1* control and CKO chromosome spread. Lower panel, distribution plot of RPA foci in early zygotene (n = 15), late zygotene (n = 18) and pachytene (n = 48 i) spermatocytes. For A-C, cells from two individuals per genotype were measured. Bars represent mean ± SD. Scale bars, 10 μm. (D) Immunostaining of SYCP3 and γH2AX on adult *Cxxc1* het and CKO chromosome spreads. Green, SYCP3; magenta, γH2AX. Scale bars, 10 μm. (E) Spermatocyte stage proportion in adult *Cxxc1* het and CKO (n = 1,066 from two individuals) spermatocytes based on SYCP3/SYCP1/γH2AX staining. *p* = 0.7 by *Chi-square* test. (F) Immunostaining of SYCP3 and SYCP1 on adult *Cxxc1* het and CKO chromosome spreads. Green, SYCP3; orange, SYCP1. Scale bars, 10 μm.
(TIF)

S5 Fig DMC1 ChIP-seq does not detect any changes in DSB number and frequencies for both PRDM9-dependent and PRDM9-independent sites in *Cxxc1* CKO. (A) Plots of activity of DSBs in two replicates of *Cxxc1* CKO and control DMC1 ChIP-seq samples. Correlation coefficient *r* = 0.96 in het controls, *r* = 0.99 in CKO samples. (B) Plot of activity of DSBs from *Cxxc1* CKO and control spermatocytes. Black dots, PRDM9-dependent sites; yellow dots, PRDM9-independent sites.
(TIF)
