# Supplementary figures and images for: CXXC1 is not essential for normal DNA double-strand break formation and meiotic recombination in mouse
Source: PLoS Genet. 2018 Oct 26;14(10):e1007657. doi: 10.1371/journal.pgen.1007657 (PMC6221362; doi:10.1371/journal.pgen.1007657)

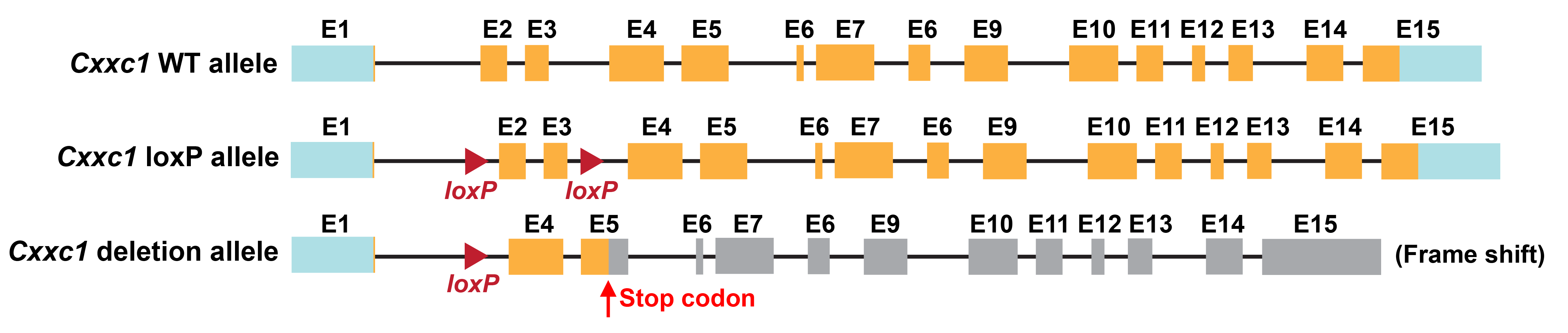

Supplement: S1 Fig — Top line, wild type allele of Cxxc1; middle line, loxP allele of Cxxc1 by flanking exon 2 and 3 with loxP sites; bottom line, deletion allele of Cxxc1 after crossing the Cxxc1loxP/loxP mice with Cre mice. Orange boxes, coding regions; blue boxes, 3 or 5 prime untranslated regions; black lines, intron regions; red triangles, loxP sites; red arrow, a stop codon generated by frame shift in the deletion allele; grey boxes, untranslated regions after stop codon in the deletion allele. Exon numbers are indicated as E1 to E15. (TIF) [file pgen.1007657.s001.tif]

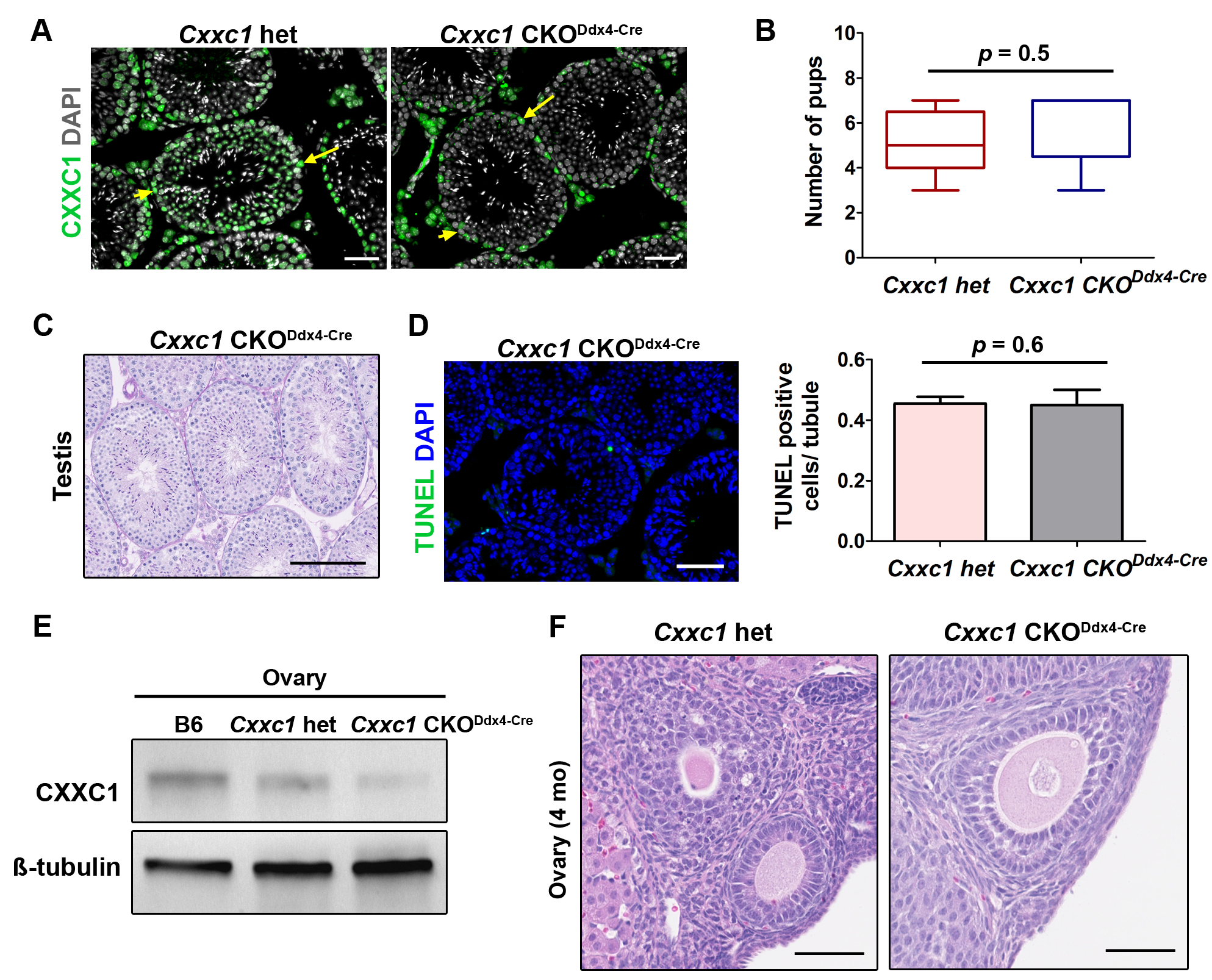

Supplement: S2 Fig — (A) Immunostaining of CXXC1 on Cxxc1 het and CKODdx4-Cre seminiferous tubule cross sections. Green, CXXC1; grey, DAPI. Long arrows, Sertoli cells; short arrows, spermatogonia. Scale bar, 50 μm. (B) Fertility tests in Cxxc1 het and CKODdx4-Cre mice. The number of viable pups was shown. (C) PAS stating of seminiferous tubules in CKODdx4-Cre. Scale bar, 100 μm. (D) TUNEL staining (left) and quantification of apoptotic germ cells (right) in Cxxc1 CKODdx4-Cre. Scale bar, 50 μm. Data represented as mean ± SD, p = 0.94 by Student t-test.(E) Western blot of CXXC1 with adult B6, Cxxc1 het and CKODdx4-Cre whole ovary extract. ß-tubulin was used as internal loading control. (F) H&E staining of secondary follicles in 4-month old Cxxc1 het and CKODdx4-Cre. Scale bar, 50 μm. (TIF) [file pgen.1007657.s002.tif]

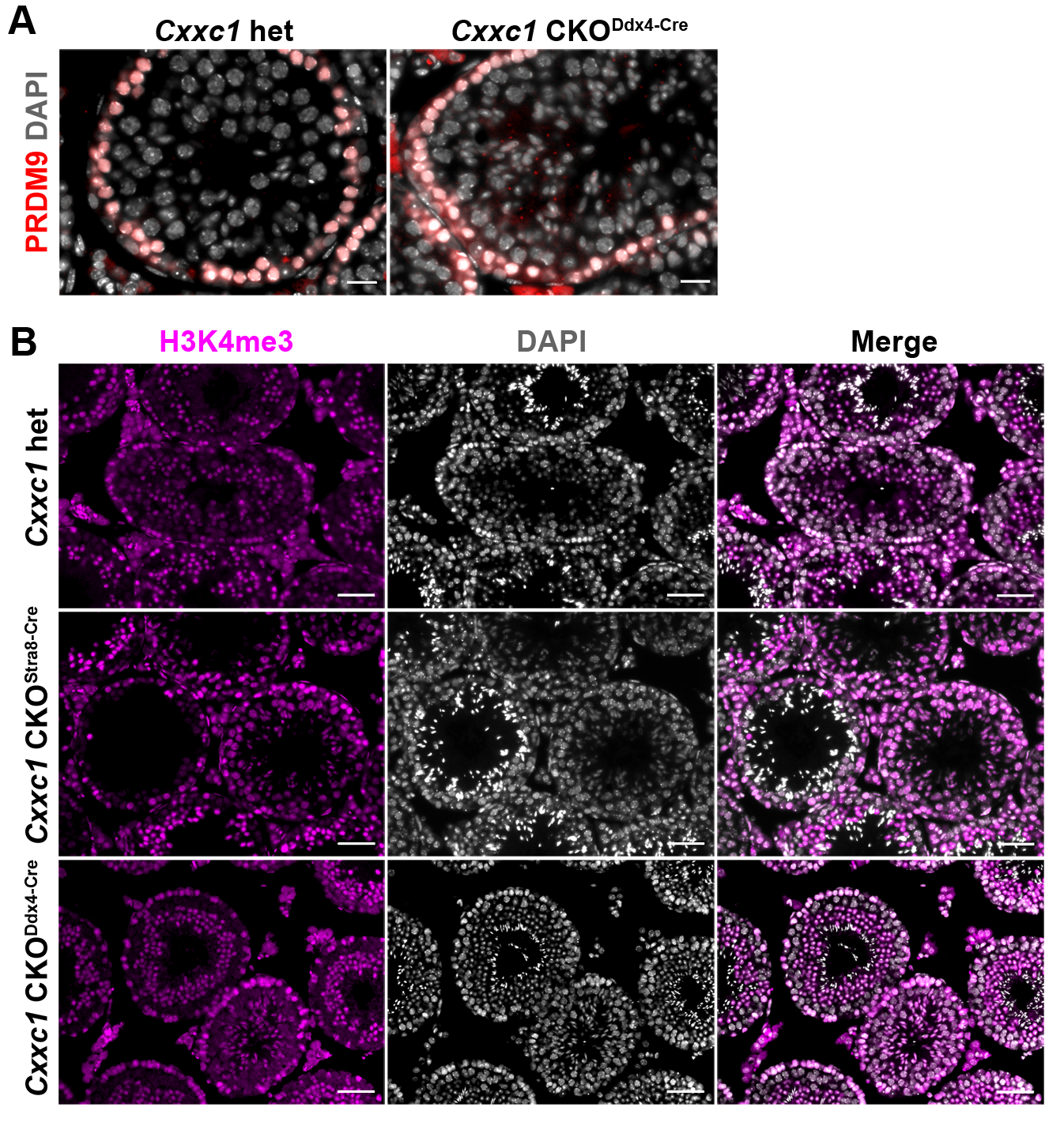

Supplement: S3 Fig — (A) Immunostaining of PRDM9 in Cxxc1 het and CKODdx4-Cre. Red, PRDM9; grey, DAPI. Scale bar, 20 μm. (B) Immunofluorescence staining of H3K4me3 on adult Cxxc1 het and CKO with Stra8- and Ddx4-Cre seminiferous tubule cross sections. Magenta, H3K4me3; gray, DAPI. Scale bars: 50 μm. (TIF) [file pgen.1007657.s003.tif]

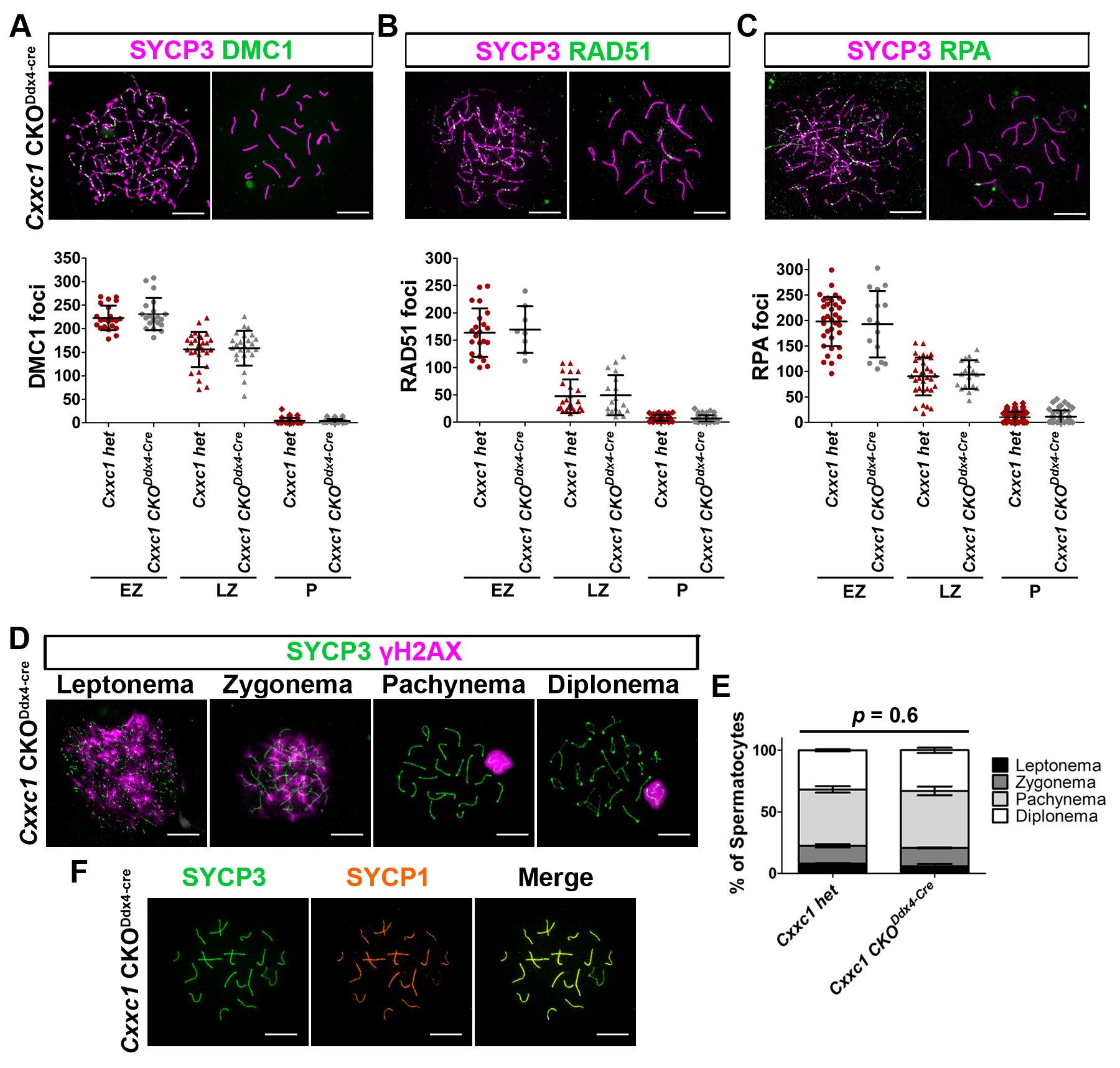

Supplement: S4 Fig — The DSB number was determined by three markers reflecting different stages of their processing. (A) DMC1 staining on Cxxc1 control and CKO chromosome spread. Lower panel, distribution plot of DMC1 foci in early zygotene (n = 20for each genotype), late zygotene (n = 25) and pachytene (n = 34) spermatocytes. (B) RAD51 staining on Cxxc1 control and CKO chromosome spread. Lower panel, distribution plot of RAD51 foci in early zygotene (n = 8), late zygotene (n = 18) and pachytene (n = 53) spermatocytes. (C) RPA staining on Cxxc1 control and CKO chromosome spread. Lower panel, distribution plot of RPA foci in early zygotene (n = 15), late zygotene (n = 18) and pachytene (n = 48 i) spermatocytes. For A-C, cells from two individuals per genotype were measured. Bars represent mean ± SD. Scale bars, 10 μm. (D) Immunostaining of SYCP3 and γH2AX on adult Cxxc1 het and CKO chromosome spreads. Green, SYCP3; magenta, γH2AX. Scale bars, 10 μm. (E) Spermatocyte stage proportion in adult Cxxc1 het and CKO (n = 1,066 from two individuals) spermatocytes based on SYCP3/SYCP1/γH2AX staining. p = 0.7 by Chi-square test. (F) Immunostaining of SYCP3 and SYCP1 on adult Cxxc1 het and CKO chromosome spreads. Green, SYCP3; orange, SYCP1. Scale bars, 10 μm. (TIF) [file pgen.1007657.s004.tif]

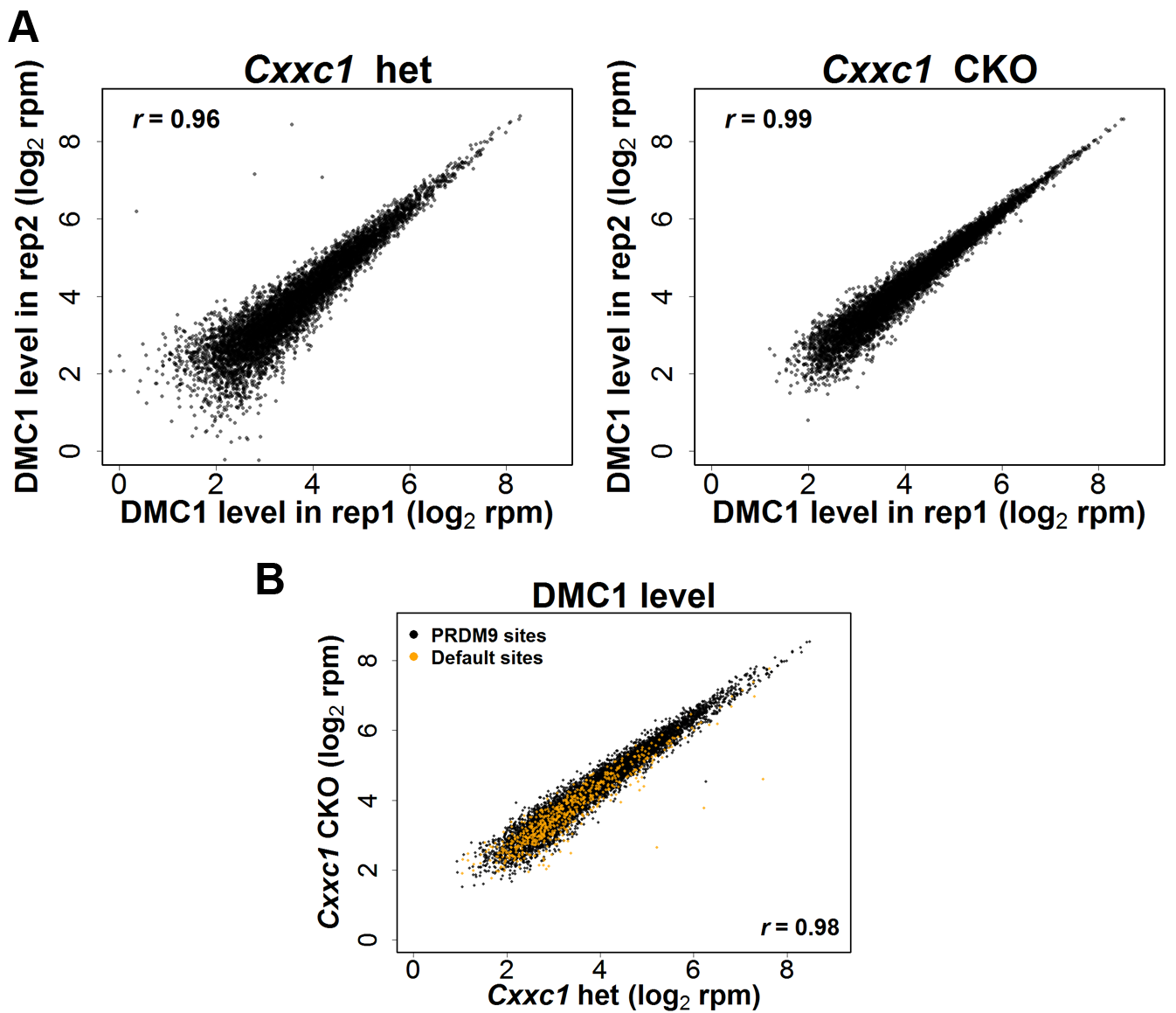

Supplement: S5 Fig — (A) Plots of activity of DSBs in two replicates of Cxxc1 CKO and control DMC1 ChIP-seq samples. Correlation coefficient r = 0.96 in het controls, r = 0.99 in CKO samples. (B) Plot of activity of DSBs from Cxxc1 CKO and control spermatocytes. Black dots, PRDM9-dependent sites; yellow dots, PRDM9-independent sites. (TIF) [file pgen.1007657.s005.tif]
